# The effect of dipeptidyl peptidase IV on disease-associated microglia phenotypic transformation in epilepsy

**DOI:** 10.1186/s12974-021-02133-y

**Published:** 2021-05-11

**Authors:** Zhicheng Zheng, Peiyu Liang, Baohua Hou, Xin Lu, Qianwen Ma, Xiaomin Yu, Song Han, Biwen Peng, Taoxiang Chen, Wanhong Liu, Jun Yin, Xiaohua He

**Affiliations:** 1 Department of Pathophysiology, School of Basic Medical Sciences, Wuhan University, Donghu Road No. 185, Wuchang, Wuhan, 430071 China; 2 Medical College, Henan Polytechnic University, Jiaozuo, China; 3 Hubei Provincial Key Laboratory of Developmentally Originated Disease, School of Basic Medical Sciences, Wuhan University, Wuhan, China; 4 Hubei Province Key Laboratory of Allergy and Immunology, School of Basic Medical Sciences, Wuhan University, Wuhan, China

**Keywords:** Disease-associated microglia, Dipeptidyl peptidase IV, Epilepsy, Microglia

## Abstract

**Background:**

Accumulating evidence suggests that disease-associated microglia (DAM), a recently discovered subset of microglia, plays a protective role in neurological diseases. Targeting DAM phenotypic transformation may provide new therapeutic options. However, the relationship between DAM and epilepsy remains unknown.

**Methods:**

Analysis of public RNA-sequencing data revealed predisposing factors (such as dipeptidyl peptidase IV; DPP4) for epilepsy related to DAM conversion. Anti-epileptic effect was assessed by electroencephalogram recordings and immunohistochemistry in a kainic acid (KA)-induced mouse model of epilepsy. The phenotype, morphology and function of microglia were assessed by qPCR, western blotting and microscopic imaging.

**Results:**

Our results demonstrated that DPP4 participated in DAM conversion and epilepsy. The treatment of sitagliptin (a DPP4 inhibitor) attenuated KA-induced epilepsy and promoted the expression of DAM markers (Itgax and Axl) in both mouse epilepsy model in vivo and microglial inflammatory model in vitro. With sitagliptin treatment, microglial cells did not display an inflammatory activation state (enlarged cell bodies). Furthermore, these microglia exhibited complicated intersections, longer processes and wider coverage of parenchyma. In addition, sitagliptin reduced the activation of NF-κB signaling pathway and inhibited the expression of *iNOS*, *IL-1β*, *IL-6* and the proinflammatory DAM subset gene *CD44*.

**Conclusion:**

The present results highlight that the DPP4 inhibitor sitagliptin can attenuate epilepsy and promote DAM phenotypic transformation. These DAM exhibit unique morphological features, greater migration ability and better surveillance capability. The possible underlying mechanism is that sitagliptin can reduce the activation of NF-κB signaling pathway and suppress the inflammatory response mediated by microglia. Thus, we propose DPP4 may act as an attractive direction for DAM research and a potential therapeutic target for epilepsy.

**Supplementary Information:**

The online version contains supplementary material available at 10.1186/s12974-021-02133-y.

## Background

Microglia are resident myeloid cells of the central nervous system (CNS) that have different developmental origins, morphological features and functions from peripheral macrophages [1–3]. Microglial phenotypic change plays a critical role in the pathogenesis of CNS diseases [4]. Microglia are traditionally classified into the classical activation phenotype (M1) and alternative activation phenotype (M2), and this process of phenotypic transformation is called polarization [5, 6]. However, recent studies have suggested that the polarization hypothesis is likely overly simplistic [7, 8]. Moreover, recent evidence shows that M1 and M2 phenotypes have not been found in vivo [9]. Technological progress in single-cell sequencing and imaging systems is leading us into a new era regarding our understanding of microglia. Using single-cell RNA-sequencing (scRNA-seq) technology, Keren-Shaul et al. identified a special microglial phenotype called disease-associated microglia (DAM) in neurodegenerative diseases [10–12]. Furthermore, DAM phenotype was detected in other diseases such as lysosomal storage disease [13], PM2.5-related disease [14] and HIV infection [15]. Accumulating evidence suggests that DAM may act as a beneficial microglial phenotype in many CNS diseases [4]. The primary purpose of recent studies in the field of DAM research was to answer two major questions [4]: (1) Is DAM a widespread phenomenon in neurological diseases? (2) What is the underlying molecular mechanism?

Abnormal discharges from neurons may activate microglia [16] and initiate the conversion of DAM [4]. However, transcriptomics analysis of DAM in epilepsy has not been reported. Microglia in lipopolysaccharide (LPS)-induced inflammatory mice show similarity to DAM, but not identical [17, 18]. Recurrent abnormal discharges from neurons can induce inflammation [19, 20], which suggests that some factors prevent DAM phenotypic transformation in epilepsy or inflammation. Here we made a comprehensive analysis of the Gene Expression Omnibus (GEO) database and found that dipeptidyl peptidase IV (DPP4) was associated with DAM phenotypic transformation in epilepsy. Furthermore, our previous research has demonstrated a positive relationship between DPP4 and epileptic seizures [21, 22]. DPP4 is a cell surface glycoprotein widely expressed in microglia, neurons and astrocytes [23]. DPP4 in microglia is strongly influenced by the surrounding microenvironment, which indicates that microglial DPP4 plays a role in the early stages of disease [24].

Furthermore, Rangaraju et al. proposed the concept of DAM subset and reported potential regulatory pathways in subset distribution such as NF-κB and RelA [9]. Consistent with the connectivity map analysis [9], some evidence suggests that inhibition of NF-κB signaling pathway may promote DAM transformation [12, 25]. The DPP4 inhibitors have shown the effect of anti-inflammation [26], further suggesting DPP4 as a regulator of DAM. Thus, this study aims to determine whether DPP4 influence DAM phenotypic transformation in epilepsy and try to explore the potential underlying mechanisms.

## Methods and materials

### Mice

C57BL/6J male mice aged 7 weeks (22 ± 1 g body weight) were obtained from Wuhan University Center for Animal Experiment/ABSL-3 Laboratory. The animals were housed at 20 ± 2 °C with 60 ± 5% humidity and a 12-h dark/light cycle. All mice had ad libitum access to standard mouse chow and water for the duration of the experiment.

### Mouse model and drug administration

Mice were randomly grouped (each group contained at least 6 mice) and were treated as follows: mice in kainic acid + sitagliptin (KA + Sita) group received sitagliptin (30 mg/kg/d i.p., Sigma, USA) for 7 days before KA injection and mice in control (Ctrl) group and kainic acid (KA) group received an equal volume of saline for 7 days.

The animal model of epilepsy was established according to a previous method [27–29]. On the last day of the sitagliptin or saline treatment, a lateral ventricle injection operation was performed. All mice were anaesthetized under 2–3% isoflurane. The right lateral brain ventricle (AP = – 0.20 mm, ML = 1.00 mm, DV = – 2.40 mm, the anterior fontanelle as the origin) was localized with a stereotactic instrument. The mice were treated as follows: Ctrl group received saline (5 μl) and KA and KA + Sita groups received kainic acid (10 μg in 5 μl i.c.v., MCE, USA). After the operation, skin was sutured, and the mice were kept under a warming place until they woke up.

### Tissue collection and sectioning

Forty-eight hours after lateral ventricle injection, the mice were anaesthetized using isoflurane and then sequentially intracardially perfused with saline and paraformaldehyde (4%, 30 ml). The mouse brain was rapidly removed and processed for paraffin embedding or frozen sections. For frozen sections, brains were post-fixed in 4% paraformaldehyde overnight and were cryoprotected using 30% sucrose at 4 °C for 48 h. After freezing in dry ice-chilled isopentane and embedding in optimal cutting temperature compound, brains were sectioned into 20-μm coronal slices using a cryostat. Sections were stored at – 80 °C before immunofluorescence staining. For paraffin sections, fixed brains were dehydrated, embedded in paraffin and were sectioned at 4-μm thickness. Sections were stored at room temperature before Nissl and haematoxylin–eosin (HE) staining.

### Microglia culture and drug administration

Primary microglia were separated from primary mixed glial cultures prepared from newborn C57BL/6J mice as previously described [30]. The BV2 microglial cell line was purchased from Shanghai Cell Research Center (Shanghai, China). Both BV2 cells and primary microglia were cultured in a humidified 5% CO_2_ incubator at 37 °C. Cells were cultured in DMEM-F12 media (Gibco, USA) supplemented with 10% fetal bovine serum (Gibco, USA) and a penicillin-streptomycin solution (Biosharp, China). The BV2 cells were passaged according to standard procedures.

The cells were plated at the proper density in plates or confocal dishes with serum-free culture medium before the experiment. Unless otherwise mentioned, experiments were performed as follows: lipopolysaccharide (LPS) group was stimulated with 100 ng/ml LPS for 24 h, and lipopolysaccharide + sitagliptin (LPS + Sita) group was pre-treated with 6 mg/ml sitagliptin for 2 h and then treated with 100 ng/ml LPS (still with 6 mg/ml sitagliptin) for 24 h. To observe p65 nuclear translocation after LPS stimulation, cells were fixed with 4% paraformaldehyde 45 min after the expose to LPS.

### Wound healing assays

Cell migration was assessed using scratch/wound healing assays. Two hours after sitagliptin treatment, straight scratches of consistent width were made in the cell monolayer using a 200-μl pipette tip, and the monolayers were washed twice with phosphate buffer saline (PBS) to remove debris. Images were captured immediately after wound generation using an inverted phase contrast microscope. Twenty-four hours later, the same demarcated area was imaged, and the wound area was calculated with ImageJ software.

### Electroencephalogram (EEG) recordings

C57BL/6J mice in the three groups were treated as described above (each group contained at least 6 mice). After the mouse was anaesthetized by isoflurane, the head was secured using stereotaxic instruments with a mouse adapter. Then, electrodes were implanted in the bilateral hippocampus (AP = – 2.30 mm, ML = ± 2.1 mm, DV = – 2.0 mm, with the anterior fontanelle as the origin). Finally, the mice were placed under a warming place until they woke up. After the same recovery period, LabChart 8 software (AD Instruments, UK) was used to collect EEG recordings for each mouse (at least 30 min per mouse) and analyze the EEG data. All EEG recordings were sampled at 10 kHz with a high-pass filter at 1.0 Hz and a low-pass filter at 1 kHz.

### Nissl and HE staining

Paraffin sections were dewaxed with xylene and rehydrated in an ethanol gradient. For Nissl staining, sections were stained with a 1% toluidine blue solution (Boster Biotech, China) and rinsed with double-distilled water. For HE staining, sections were immersed in haematoxylin solution for 3 min, rinsed three times with water, soaked in a hydrochloric acid alcohol solution for 5 s, and soaked in eosin solution for 2 min. Finally, all sections were dehydrated through a graded ethanol series to xylene and then cover-slipped with Permount.

### Reverse transcriptase quantitative PCR (RT-qPCR)

TRIzol reagent (Invitrogen, USA) was used to extract total RNA from the mouse hippocampus or microglia. Then, reverse transcription was performed using the Revert Aid First Strand cDNA Synthesis Kit (Thermo Scientific, USA) according to the manufacturer’s protocol. RT-qPCR and data analysis were performed with a Bio-Rad CFX96 Real-time PCR system (Bio-Rad Laboratories, Hercules, CA, USA). Each 20 μl reaction contained 10 μl SYBR Green (Aidlab Biotech, China), 0.8 μl of each primer, 2 μl cDNA and double-distilled water. A complete list of primer sequences is provided in supplementary Table 1. The cycling conditions were 95 °C for 30 s, followed by 40 cycles of 95 °C for 10 s, 55 °C for 15 s and 72 °C for 20 s. The 2^–ΔΔct^ method was used to calculate mRNA expression relative to *GAPDH*.

### Western blotting

Tissues and cells were lysed on ice using radioimmunoprecipitation assay (RIPA; Biosharp, China) buffer supplemented with phenylmethanesulfonyl fluoride (PMSF; Biosharp, China), protease inhibitors (TargetMol, USA) and phosphatase inhibitors (TargetMol, USA). The total protein concentration in the supernatant of each sample was quantified using a BCA protein assay according to the manufacturer’s protocol (BCA Protein Quantification Kit, Thermo Scientific, USA). The detailed western blotting procedure was previously described [21]. Primary antibodies against the following proteins were used in the experiments: DPP4 (1:1000, Abcam), CD68 (1:1000, Abcam), ITGAX (1:1000, Proteintech), NF-κB p65 (1:1000, Cell Signaling Technology, USA), MyD88 (1:1000, Proteintech), TRAF6 (1:3000, Proteintech) and NF-κB phosphorylated p65 (p-p65; 1:1000, Cell Signaling Technology, USA). Protein expression is presented relative to the levels of β-actin (1:10000, Abcam).

### Immunofluorescence staining

Frozen sections were warmed from – 80 to – 20 °C. Then, the sections were equilibrated to room temperature and fixed with methanol. After treated with 0.5% Triton X-100 in PBS for 20 min, the samples were blocked with 10% goat serum in PBS for 1 h. Then, the samples were incubated at 4 °C overnight with primary antibodies against the following proteins: ITGAX (1:50, Abcam), IBA1 (1:200, Abcam), CD68 (1:100, Abcam) and NF-κB p65 (1:400, Cell Signaling Technology, USA). After three washes with PBS, the samples were incubated with the appropriate fluorescein-labelled secondary antibody (goat anti-mouse DyLight 488, goat anti-rabbit DyLight 594, goat anti-rat DyLight 488, Abbkine, USA) for 1 h at room temperature. Then, nuclei were stained with 4′,6-diamidino-2-phenylindole (DAPI; 1:100,000, Sigma). The TrueVIEW Autofluorescence Quenching Kit (Vector Laboratories) was used to quench autofluorescence. Finally, the anti-fluorescence quencher was used to seal all sections. The sections were observed with a Leica-LCS-SP8-STED confocal laser-scanning microscope (Leica Microsystems, Wetzlar, Germany) using appropriate laser beams and filters.

### Confocal imaging and analyzes

Confocal microscopy images were obtained using a confocal microscope. The confocal settings, including the laser power, detector gain, amplifier offset, amplifier gain and pinhole size, were unchanged during scanning. For tissue immunofluorescence, at least three images were taken per hippocampal region using a × 63 oil differential interference contrast objective at 1024 × 1024 pixel resolution, with a z-step size of 0.5 μm at 10 μm thickness. After confocal z-stack images were acquired, the mean fluorescence intensity was evaluated using LAS X software. Then, Sholl analysis was performed with ImageJ software using the plugin “Sholl analysis” (http://imagej.net/Sholl_Analysis). For cellular immunofluorescence, at least five randomly selected fields were analyzed in each sample, and the colocalization coefficients were calculated using the colocalization tool for the Leica-LCS-SP8-STED.

### Statistical analysis

For in vivo experiments, the *n* values refer to the number of individual animals in each group. For in vitro studies, the *n* values indicate the number of times the experiment was independently replicated. The data are presented as the mean ± standard error of the mean (SEM). Normality was tested using the Shapiro–Wilk test. Unless specifically mentioned, the statistical significance of differences between test groups was evaluated using one-way analysis of variance (ANOVA) and Tukey’s multiple comparison test, while the Mann–Whitney test was used for datasets that did not pass normality test. *P* < 0.05 was considered to indicate statistical significance.

## Results

### Inhibition of DPP4 attenuates KA-induced epilepsy

**Fig. 1:**
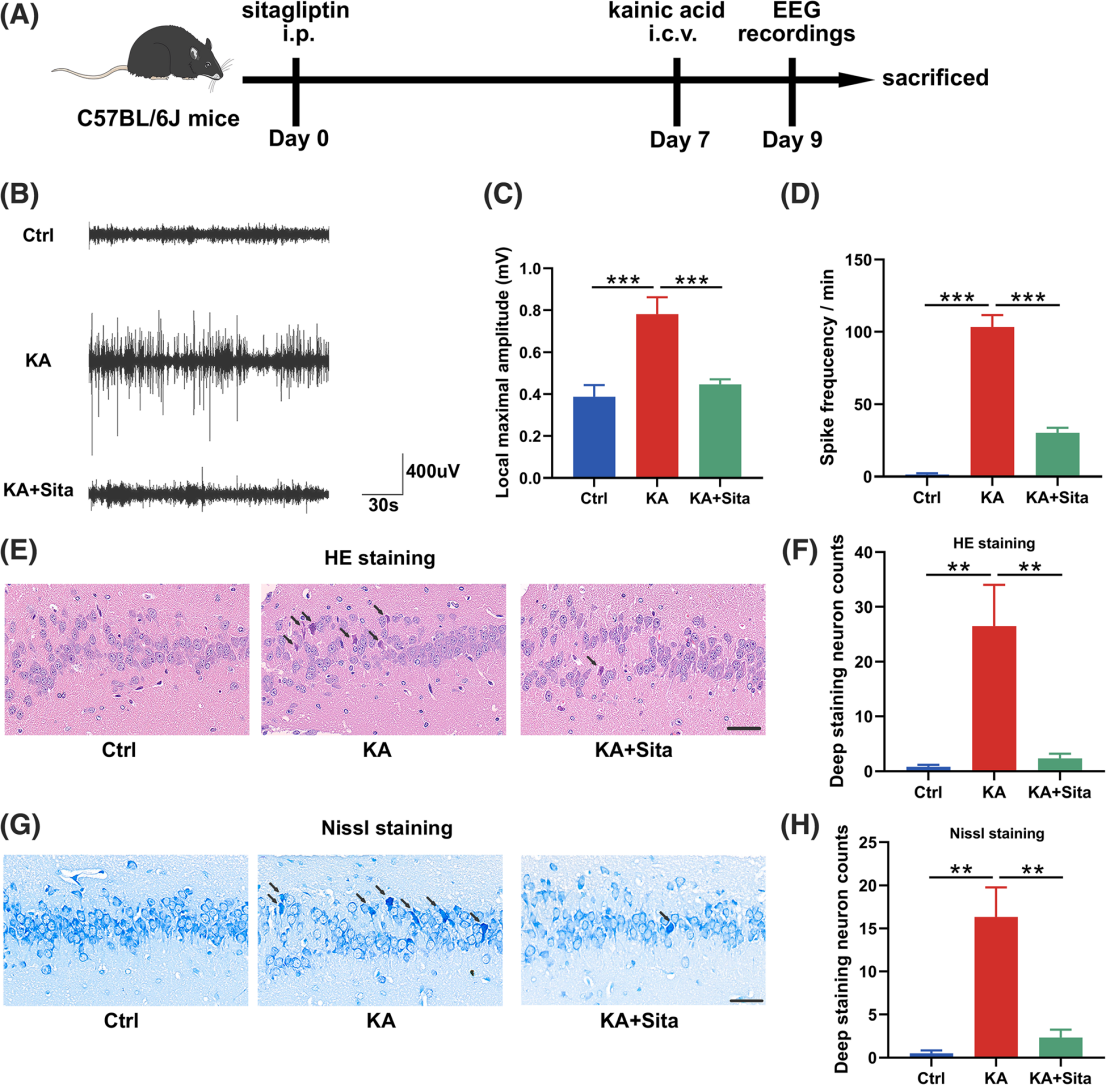
Inhibition of DPP4 attenuates KA-induced epilepsy. **a** Flowchart of the experiment in vivo. **b** Representative EEG traces of electrographic activity in control (*n* = 6), kainic acid (*n* = 9), and kainic acid + sitagliptin (*n* = 10) groups. **c** The local maximum amplitude and **d** spike frequency in EEG data were calculated by LabChart 8 software. **e**, **g** Representative HE and Nissl staining of the hippocampal region. Black arrows indicate damaged neurons. Scale bar = 40 μm. **f**, **h** The number of damaged neurons for each group was counted at high magnification (*n* = 5–6 per group). Data are presented as the mean ± SEM. **P < 0.01, ***P < 0.001

To search for potential targets involved in DAM conversion, we collected both up- and downregulated gene profiles of epileptic hippocampus and gene-edited microglia from four independent GEO datasets (Supplementary Figure 1A; Supplementary Table 2). Intersection analysis identified three genes with consistent patterns of change: *Dpp4*, *ceruloplasmin* (*Cp*) and *periostin* (*Postn*; Supplementary Figure 1B). *Dpp4* and *Cp* are upregulated among four groups (Supplementary Figure 1C). To observe the changes in epilepsy treated with DPP4 inhibitor sitagliptin, we evaluated epileptic seizures by EEG recordings (Fig. 1a). The EEG traces showed that sitagliptin attenuated KA-induced epileptic seizures, and the trace in KA + Sita group had a lower local maximal amplitude and reduced spike frequency (Fig. 1b–d). Next, we investigated neuronal loss in the hippocampus, which is the KA susceptible area [31]. Triangulated pyknotic nuclei and cytoplasmic shrinkage (Fig. 1e, g) were found in the hippocampal neuron in KA groups, whereas KA-induced neuronal loss was relieved by sitagliptin treatment (Fig. 1f, h). Taken together, sitagliptin ameliorated KA-induced neurological damage and attenuated KA-induced epileptic seizures.

### Inhibition of DPP4 alters the activation state of microglia in epileptic mice

**Fig. 2:**
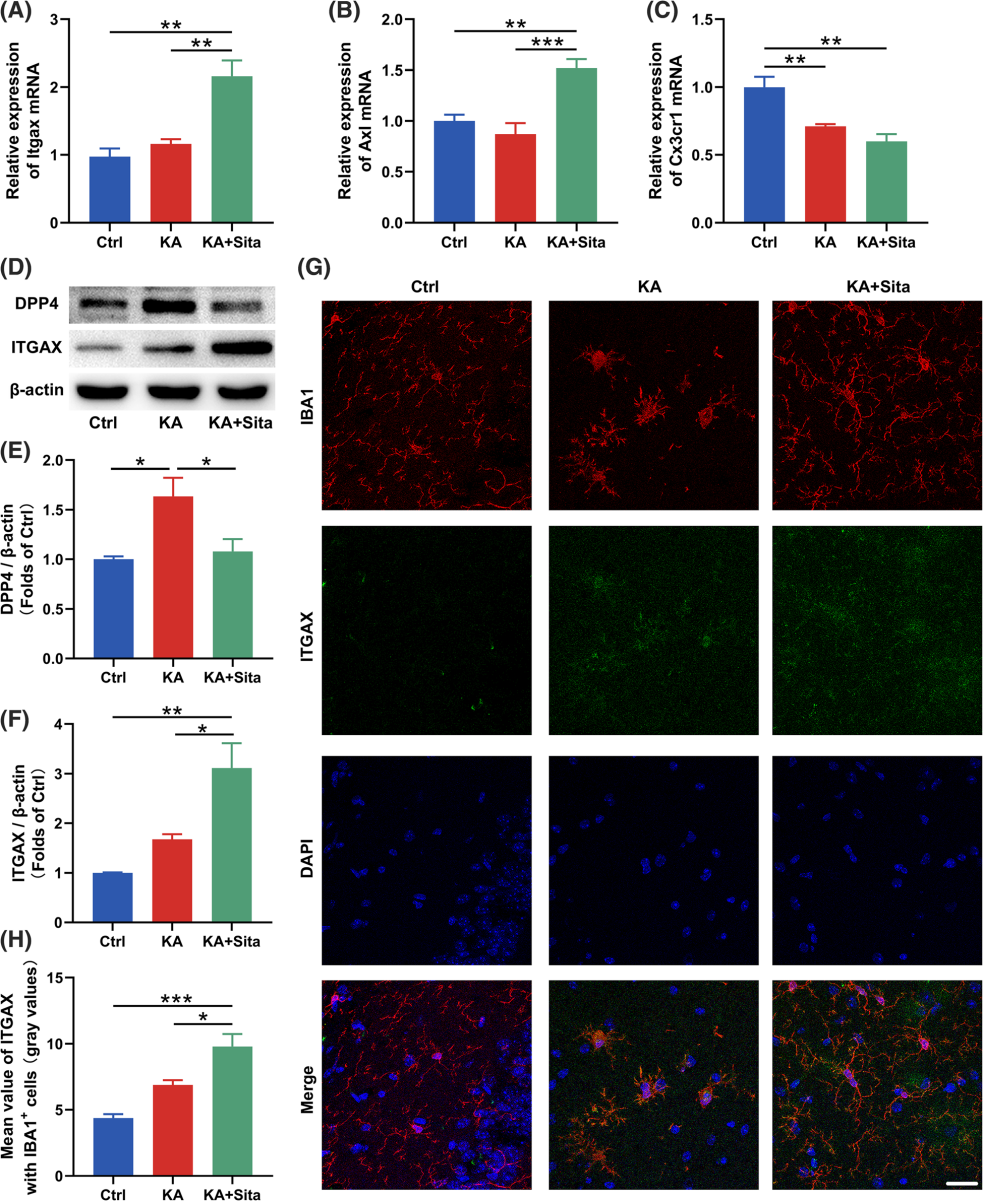
DPP4 participates in DAM phenotypic transformation in epileptic mice. **a**–**c** Gene expression in hippocampus from mice treated with kainic acid or kainic acid + sitagliptin (*n* = 6–7 per group). Selected markers: DAM: *Itgax* and *Axl*; homeostatic: *Cx3cr1*. **d**–**f** Representative western blotting images and statistical analysis of DPP4 and ITGAX in the hippocampus (*n* = 4). β-Actin was used as a loading control. **g** Representative overlay images of IBA1/ITGAX/DAPI staining in the hippocampus (IBA1, red; ITGAX, green; DAPI, blue). Scale bar = 25 μm. **h** The statistical analysis of ITGAX in IBA1^+^ microglia is shown on the left side. Data are expressed as the mean ± SEM. *P < 0.05, **P < 0.01, ***P < 0.001

Previous research revealed some DAM markers [4, 10], and here we found that sitagliptin promoted the expression of DAM genes (*Itgax* and *Axl*) in mice from the KA group (Fig. 2a, b). Besides the gene marker, we found that the microglial homeostatic gene *Cx3cr1* was significantly decreased in the KA + Sita group compared to the Ctrl group (Fig. 2c). Western blotting showed that sitagliptin attenuated KA-induced upregulation of DPP4 and promoted ITGAX expression (Fig. 2d–f). These data indicated that microglia might convert into DAM when DPP4 was inhibited. Notably, there was an increasing tendency of ITGAX in the KA group compared to the Ctrl group. There was no significant difference observed between the Ctrl group and the KA group using ANOVA and Tukey’s multiple comparison test (Fig. 2f).

**Fig. 3:**
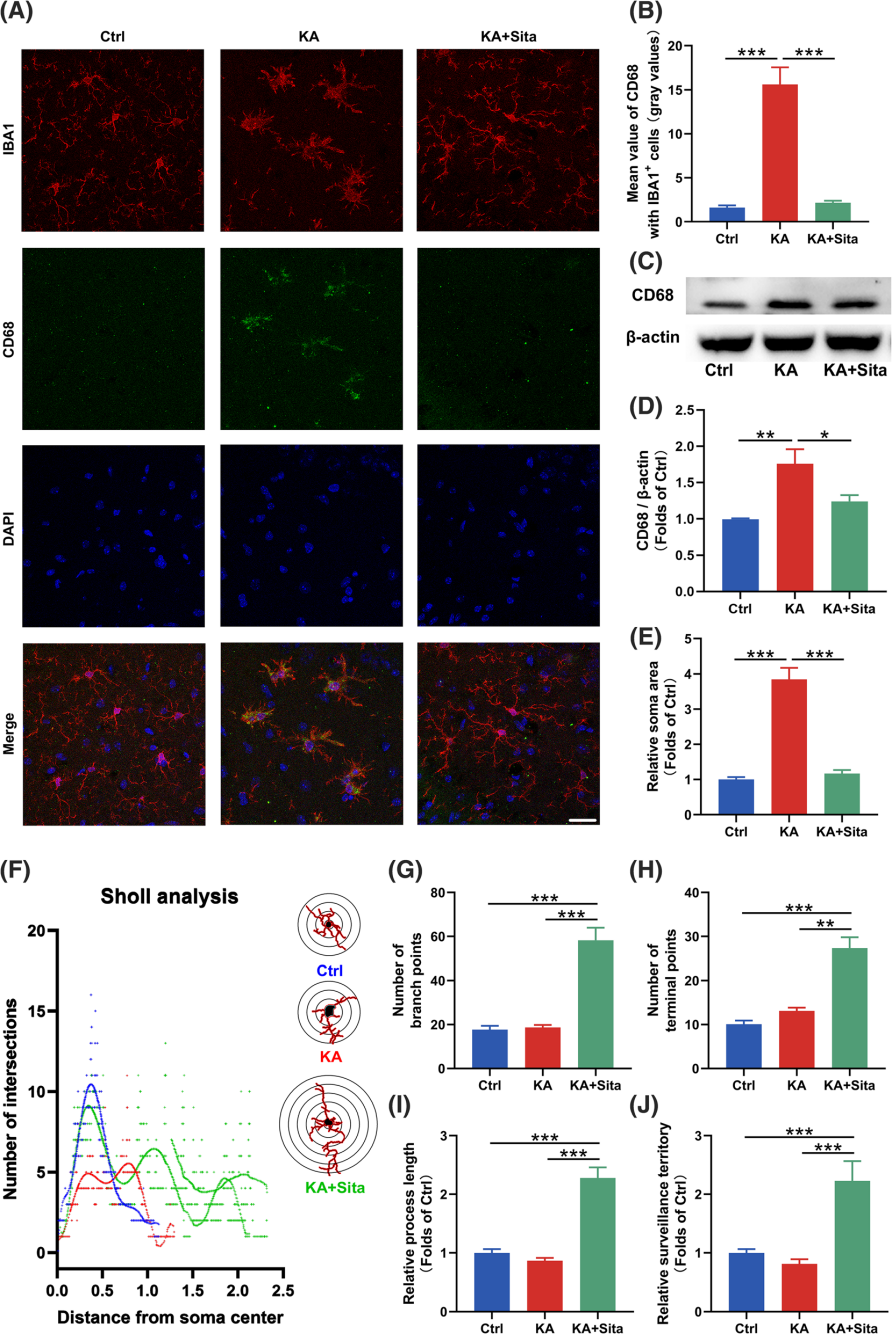
Inhibition of DPP4 alters the activation state of microglia in epileptic mice. **a** Representative overlay images of IBA1/CD68/DAPI staining in the hippocampus of the three groups (IBA1, red; CD68, green; DAPI, blue). Scale bar = 25 μm. **b** The statistical analysis of CD68 mean value in IBA1^+^ microglia. **c**, **d** Western blotting for CD68 in the hippocampus. Relative CD68 expression was normalized to β-actin (*n* = 3). **e** The soma area, **f** Sholl intersections, **g** process branch points, **h** terminal points and **i** the relative length of processes of IBA1^+^ microglia in the hippocampus of the three groups (*n* = 12–18 cells in 3 mice per group). **j** The average area covered by individual IBA1^+^ microglia in each group were calculated by LAS X software (*n* = 12–18 cells in 3 mice per group). Data are presented as the mean ± SEM. **P < 0.01, ***P < 0.001

To further investigate the changes of microglia, merged high-resolution confocal images were analyzed. Colocalization analysis showed that ITGAX expression was increased in hippocampal IBA1^+^ microglia with the sitagliptin treatment (Fig. 2g, h). CD68, the microglial inflammation marker, was upregulated in the KA group, but was significantly downregulated in the KA + Sita group (Fig. 3a, b). Western blotting also revealed that the expression of CD68 was increased in epileptic mice, while this increase was inhibited by sitagliptin treatment (Fig. 3c, d). Hippocampal IBA1^+^ microglia with sitagliptin treatment did not exhibited the inflammatory activation state (enlarged cell bodies [32]; Fig. 3a, e). Interestingly, according to the Sholl analysis, microglia in the KA + Sita group had the most complicated intersections (Fig. 3f–h) and the longest cell processes (Fig. 3i) among the three groups. Brain parenchymal area covered by single microglia suggests surveillance capability of microglia. The area in the KA + Sita group was significantly larger than the other two groups (Fig. 3j). Altogether, microglia in epileptic mice treated with sitagliptin exhibited another activated state that differs from KA-stimulated microglia, and these microglia might represent DAM.

### Inhibition of DPP4 alters the activation state of microglia in vitro

**Fig. 4:**
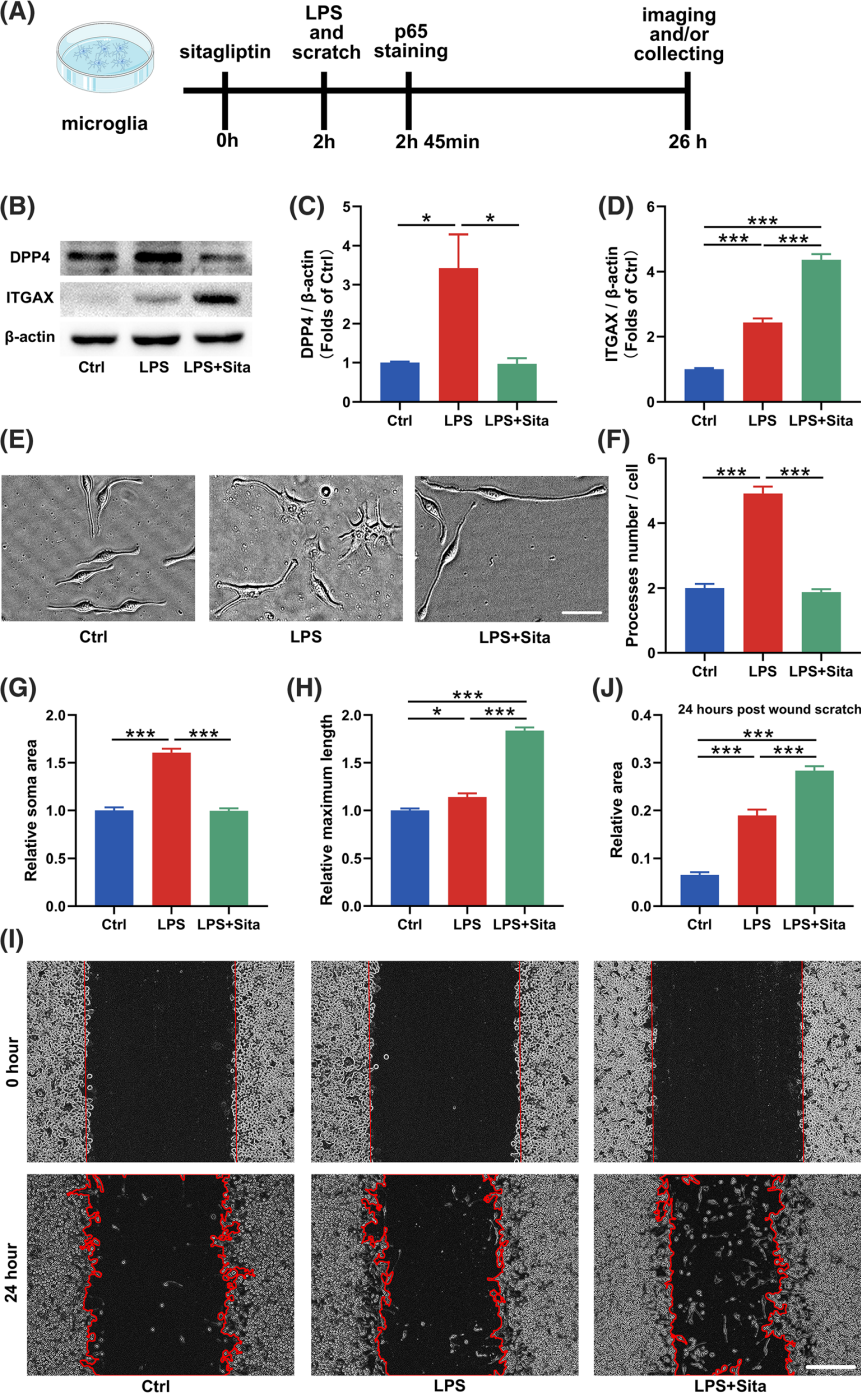
Inhibition of DPP4 alters microglial morphology and function in vitro. **a** Flowchart of the experiment in vitro. **b**–**d** Whole-cell lysates were subjected to western blotting to examine the protein levels of ITGAX and DPP4 in BV2 microglial cells after treatment with LPS or LPS + sitagliptin. Data were normalized to β-actin (*n* = 3–4). **e** Representative morphology of BV2 microglia in each group under a phase-contrast microscope. Scale bar = 50 μm. **f** Statistics of processes number, **g** soma area and **h** maximum length in BV2 microglia (*n* = 20–24 cells per group). **i**, **j** Microglial migration was measured using wound healing assays. Scale bar = 200 μm. Data are presented as the mean ± SEM. *P < 0.05, ***P < 0.001

LPS stimulation leads to inflammatory responses of macrophages and microglia, resulting in overexpression of DPP4 [33, 34]. To further explore the effect of DPP4 on DAM phenotypic transformation under inflammation, we cultured primary microglia and BV2 microglia cell line (Fig. 4a). Consistent with the in vivo results, the mRNA expression of *Itgax* and *Axl* were increased in the LPS + Sita group, while *Cx3cr1* gene expression was still lower in the LPS and LPS + Sita groups compared to the Ctrl group (Supplementary Figure 2A–C). BV2 cells exhibited the same gene expression changes (Supplementary Figure 2D–F). Western blotting demonstrated that sitagliptin inhibited the LPS-induced upregulation of DPP4 and increased ITGAX expression in BV2 cells (Fig. 4b–d). The ITGAX expression was higher in the LPS group compared with the Ctrl group (Fig. 4d). Furthermore, sitagliptin treatment decreased the LPS-induced upregulation of CD68 (Supplementary Figure 2G, H).

Next, we examined the morphology and function of BV2 cells when DPP4 were inhibited. Sitagliptin decreased the processes number and cell body size of BV2 cells treated with LPS (Fig. 4e–g). In the LPS + Sita group, besides the processes number being decreased, the maximum length of the processes was increased (Fig. 4e, h). Wound healing assays revealed that migration ability of microglia was increased when treated with LPS, and this migration ability was further enhanced by sitagliptin treatment (Fig. 4i, j). Altogether, sitagliptin-induced DAM exhibited unique morphological characteristic and function that differ from the untreated or the LPS-treated microglia.

### The NF-κB pathway is downregulated after DPP4 inhibition

**Fig. 5:**
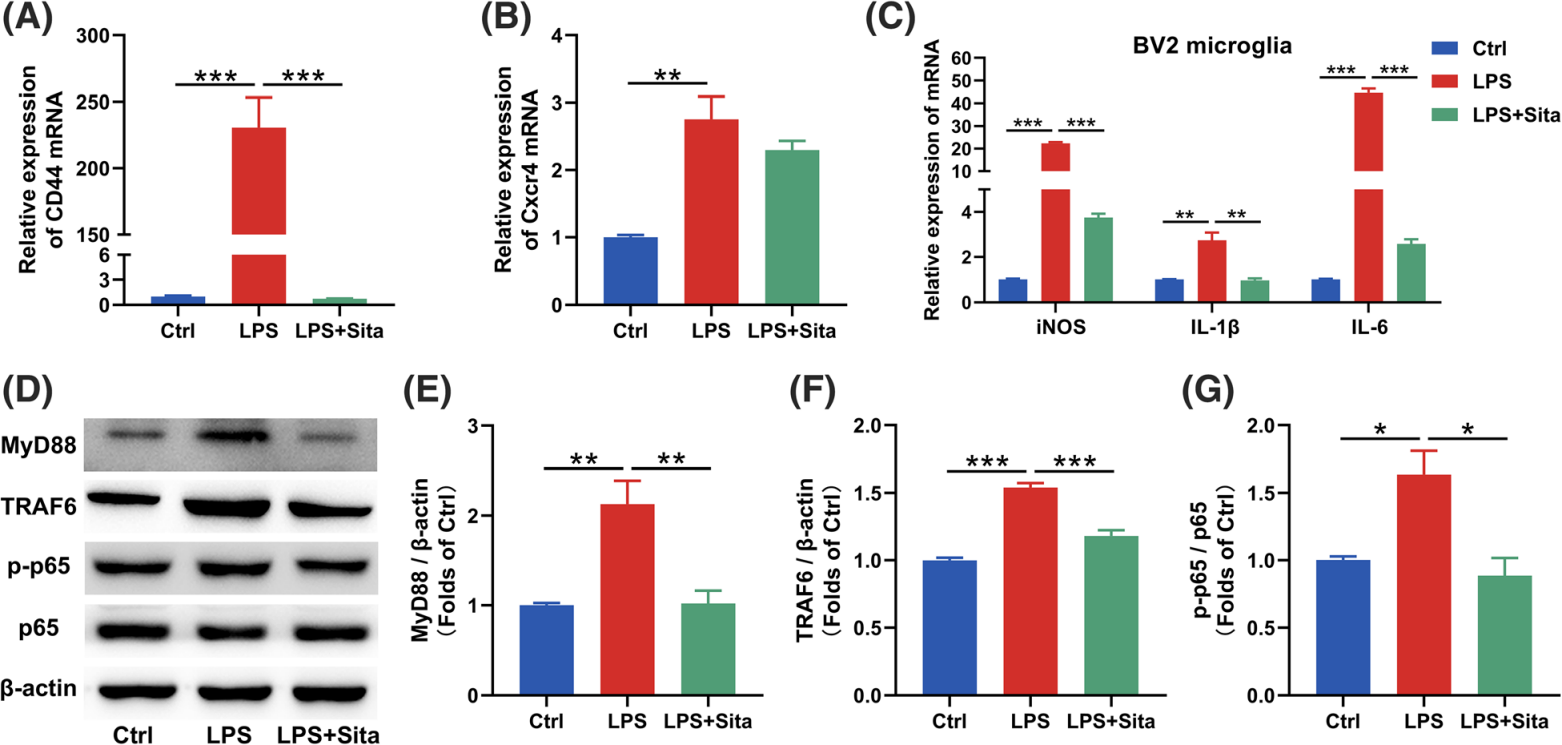
Downregulation of the NF-κB pathway after DPP4 inhibition in vitro. **a**, **b** The mRNA expression of genes associated with DAM subset was analyzed by RT-qPCR in BV2 cells. Selected markers: proinflammatory DAM: *CD44*; anti-inflammatory DAM: *Cxcr4*. **c** The mRNA expression of *iNOS*, *IL-1β* and *IL-6* in BV2 microglia. **d**–**g** Western blotting analysis of MyD88, TRAF6, p-p65 and p65 in BV2 microglia. All bands were normalized to β-actin (*n* = 3). Data are presented as the mean ± SEM. *P < 0.05, **P < 0.01, ***P < 0.001

To explore potential molecular mechanisms underlying the transformation, we detected DAM subset genes. We found that the proinflammatory gene *CD44* was upregulated when the cells were treated with LPS, and this gene was downregulated after sitagliptin treatment (Fig. 5a). As for anti-inflammatory gene *Cxcr4*, though *Cxcr4* gene was increased with the LPS treatment, the gene did not further change with sitagliptin treatment (Fig. 5b). Consistent with the DAM subset genes alterations, sitagliptin inhibited the expression of *iNOS*, *IL-1β* and *IL-6* in the LPS-treated cells (Fig. 5c). Western blotting showed that sitagliptin inhibited LPS-induced activation of the NF-κB pathway by downregulating the expression of MyD88 and TRAF6 and reducing the ratio of p-p65/p65 (Fig. 5d–g). Immunofluorescence colocalization analysis also indicated that sitagliptin inhibited the nuclear translocation of p65 (Supplementary Figure 3A–B).

**Fig. 6:**
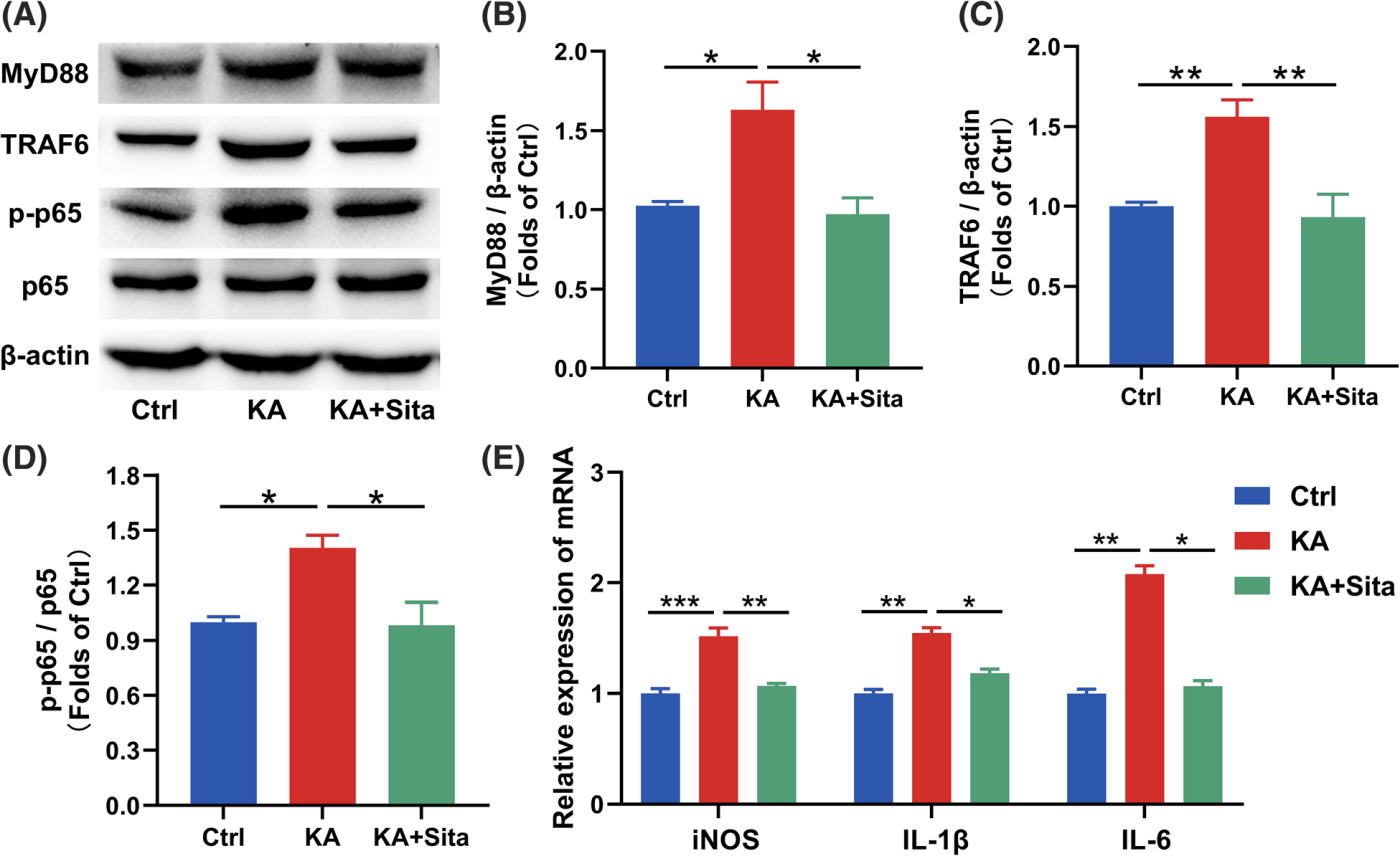
Downregulation of the NF-κB pathway after DPP4 inhibition in vivo. **a** Mouse hippocampus homogenates were subjected to western blotting. **b**–**d** Quantification analysis of MyD88, TRAF6 and p-p65/p65. Data were normalized to β-actin protein expression (*n* = 3–4). **e** The mRNA expression of *iNOS*, *IL-1β* and *IL-6* in the hippocampus. Data are presented as the mean ± SEM. *P < 0.05, **P < 0.01, ***P < 0.001

Then, we verified the inhibitory effect of sitagliptin on the NF-κB pathway in the epileptic mice model. We found that sitagliptin suppressed the KA-induced upregulation of MyD88 and TRAF6, and this suppression accompanied the reduction of p-p65 to total p65 (Fig. 6a–d). RT-qPCR results also showed that sitagliptin reduced the *iNOS*, *IL-1β* and *IL-6* expression in the hippocampus of epileptic mice (Fig. 6e).

## Discussion

In a KA-induced epilepsy model, we found that sitagliptin attenuated KA-induced epilepsy and increased the expression of ITGAX in microglia, which suggested that DPP4 inhibiting could promote DAM conversion. DAMs have two specific molecular signatures [4]: One is low expression of microglial homeostatic gene, such as Cx3cr1, while the high-expression of Itgax and Axl are considered to be another. Among these molecular markers, Itgax is regarded as the most effective candidate: “there are no cells with a DAM signature that are Itgax negative.” [10], and the Axl is a possible functional receptor for DAM [4]. As for Cx3cr1, the low expression of homeostatic gene is an obvious feature of microglial activation [35]. In our study, the Cx3cr1 expression is downregulated in the KA group and KA + Sita group, suggesting microglia was activated after KA stimulation and it might transform to another activated state with further sitagliptin treatment. This change is consistent with a previous study, while the treatment of Kv1.3 blocker ShK-223 promotes the DAM molecular signature with the downregulated homeostatic gene of microglia [9].

We found that Itgax and Axl are significantly upregulated in the KA + Sita group compared to the KA group or Ctrl group (Fig. 2f), and we also observed the increasing tendency of Itgax in the KA group compared to the Ctrl. This is concordant with a previous research, suggesting there is a tendency for microglia to transform toward DAM under injury [9, 17]. For Itgax was further increased in the KA + Sita group compared to the KA group in our experiment, we speculate that there are two needs that must be fed for DAM transformation, one is the injury situation and the other is downregulation of DPP4. Furthermore, severe or long-term disease states will perturb the DAMs, leading a more severe inflammatory conditions or neurodegenerative disorders [36]. Under these conditions, DAM exhibited characteristic gene expression of the proinflammatory subset [9]. Our present experiments indicated sitagliptin reduced the expression of CD44 (the marker of proinflammatory DAM), which suggested that it probably regulated transcriptional regulators of proinflammatory DAM (NF-κB and RelA). In fact, sitagliptin have exhibited strong anti-inflammatory effects in many disease models, such as Parkinson’s disease and LPS-induced lung injury [37, 38]. It is reported that DPP4 can activate NF-κB signaling, and DPP4 inhibitors can attenuate the activation of NF-κB signaling pathway [39, 40]. These effects were confirmed in our epileptic model that sitagliptin treatment inhibited the NF-κB signaling pathway. Overall, these findings suggest that NF-κB signaling may take part in the DAM transformation in epilepsy. On the other hand, inflammation is both a cause and consequence of epilepsy [41, 42]. Abnormally activated microglia, such as stimulated by LPS or KA, aggravate nervous system damage by secreting a variety of inflammatory factors [5, 6]. We found that sitagliptin reduced proinflammatory factors (iNOS, IL-1β and IL-6) and inflammatory marker (CD68) in epileptic hippocampus and LPS-activated microglia. These data provide further support for the hypothesis of “DPP4 over-expression promotes epilepsy” [21, 22].

Next, we used LAS X software and Sholl analysis to evaluate morphological parameters of microglia [43]. We found that activated microglia had a unique morphology and functions apart from showing specific molecular signatures. DAM may have special functions such as stronger phagocytic capacity and greater surveillance faculty [4]. In our experiment, sitagliptin-induced DAM also showed these unique characteristics. The area of brain parenchyma covered by microglia was increased with the sitagliptin treatment, which suggested greater surveillance capability as described before [43, 44]. Moreover, microglia treated with both LPS and sitagliptin exhibited stronger migration capability and elongated cell shape. Recent research has reported that the longer morphology of microglia indicates greater phagocytic ability [45, 46]. Longer processes of microglia may enhance the communication between microglia and neurons [47] and stabilize the CNS [4, 48]. These functional changes accord with DAM characteristics identified by Gene Ontology (GO) analysis [9, 49]. The evidence above suggests that the DAM induced by sitagliptin can protect and approve the function of the CNS.

It has been reported that the triggering receptor expressed on myeloid cells 2 (TREM2) signaling pathway plays an important role in DAM phenotypic transformation [10]. And DAM phenotypic transformation requires two steps [10], DAM stage 1 and DAM stage 2 [4]. TREM2 knockout in mice blocks DAM transformation from stage 1 to stage 2 [50, 51]. TREM2 expression is significantly decreased upon LPS stimulation in microglia [33]. These reports suggested that downregulation of TREM2 may act as the obstacle of DAM transformation under inflammatory conditions. Using GEO database, we found that DPP4 and Cp overexpression was associated with the dysregulation of TREM2 and epilepsy. To further verify that the overexpressed DPP4 prevents DAM phenotypic transformation, we constructed an LPS-induced inflammatory cell model and found that the expression of DAM markers was promoted by sitagliptin treatment. We also noted that the expression of Trem2 was decreased after LPS stimulation, but sitagliptin failed to rescue this decrease (data not shown). These data suggest that DPP4 may be located in the downstream of TREM2 or DPP4 can regulate DAM transformation independent of TREM2 signaling. The latest research shows that TREM2 is not the only factor regulating microglial phenotype and function [49, 50, 52]. Thus, DPP4 may be a suitable target for further studies of DAM. Using gene-editing technology can help us understand the molecular regulatory network of microglia better.

In addition, both our results and previous studies [17, 18] suggest that LPS-activated microglia are different from DAM in stage 1. LPS-activated microglia are more similar to M1-like microglia [53], for they have enlarged cell bodies and increased processes [32] and they can secret multiple inflammatory factors [6]. Hippocampal microglia in KA-treated mice also exhibited some characteristics of inflammatory activation [19, 54]. The specific difference between those inflammatory microglia and DAM in stage 1 remains unknown. We believe it is necessary to approach cell-specific sequencing approaches, such as single-cell RNA sequencing, for a more accurate classification of DAM in epilepsy in future study.

## Conclusion

We found that DPP4 overexpression on microglia hinders its protective functions. The abnormal activated microglia aggravate the progression of epilepsy. Meanwhile, inhibition of DPP4 promotes DAM phenotypic transformation in epileptic and inflammatory models, with dramatic changes in morphology and functions.

## Supplementary Information

**Additional file 1: Supplementary Figure 1**. Potential factors associate with DAM phenotypic transformation in epilepsy. (A) The differentially expressed genes (DEGs; red, upregulated genes; blue, downregulated genes) in the mouse model of epilepsy (Model, GSE1831 and GSE40490), TREM2 knockout (KO, GSE70475) group and DAP12 knockout (GSE9043) group relative to the control groups. DEGs were identified from pairwise comparisons with two selection criteria: fold change > 2.0 and corresponding adjusted *P* value < 0.05. (B) Venn diagrams of three commonly changed genes in four databases. (C) Heatmaps visualizing the mRNA expression of the three genes.

**Additional file 2: Supplementary Figure 2**. Inhibition of DPP4 alters the activation state of microglia in vitro*. (*A–C) *Itgax*, *Axl* and *Cx3cr1* mRNA expression was measured by RT-qPCR in both mouse primary microglial cells and (D–F) BV2 microglial cells. (G–H) Western blotting of CD68 protein in BV2 microglia. Relative CD68 expression was normalized to β-actin (*n* = 3). Data are presented as the mean ± SEM. *P < 0.05, **P < 0.01, ***P < 0.001.

**Additional file 3: Supplementary Figure 3**. Colocalization analysis of microglial cells. (A) Immunofluorescence staining showing that p65 colocalized with DAPI (magenta indicates a merge). (B) The statistical analysis is shown on the right side. Scale bar = 25 μm. Data are presented as the mean ± SEM. ***P < 0.001.

**Additional file 4: Supplementary Table 1**. Primer sequence.

**Additional file 5: Supplementary Table 2**. Datasets from GEO database.

## Data Availability

All data generated or analyzed during this study are included in this published article and its additional files.

## Abbreviations

DAM: Disease-associated microglia
DPP4: Dipeptidyl peptidase IV
CNS: Central nervous system
scRNA-seq: Single-cell RNA-sequencing
LPS: Lipopolysaccharide
GEO: Gene Expression Omnibus
KA: Kainic acid
Sita: sitagliptin
Ctrl: Control
HE: Haematoxylin–eosin
PBS: Phosphate buffer saline
EEG: Electroencephalogram
RT-qPCR: Reverse transcriptase quantitative PCR
SEM: Standard error of the mean
TREM2: Triggering receptor expressed on myeloid cells 2
