# Supplementary figures and images for: The effect of dipeptidyl peptidase IV on disease-associated microglia phenotypic transformation in epilepsy
Source: J Neuroinflammation. 2021 May 11;18:112. doi: 10.1186/s12974-021-02133-y (PMC8114532; doi:10.1186/s12974-021-02133-y)

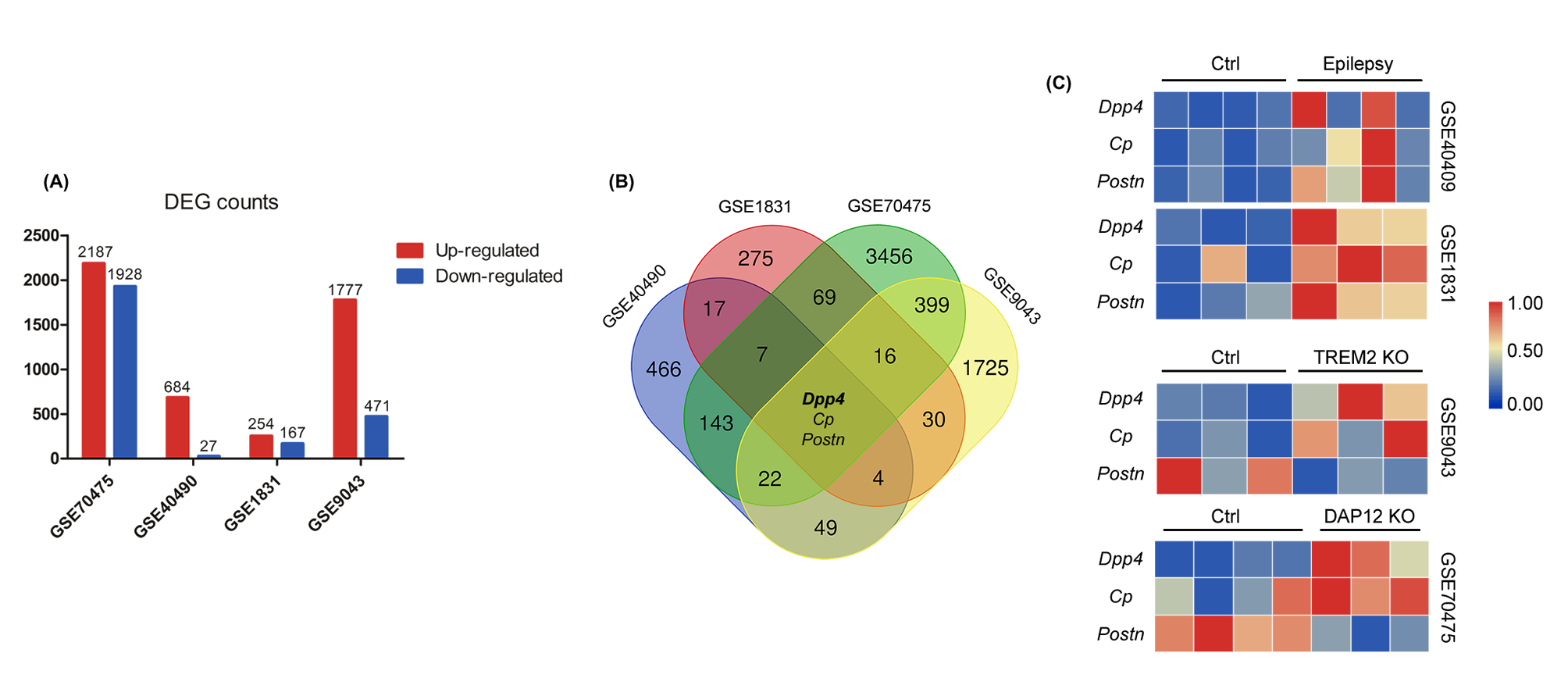

Supplement: Supplementary file 1 — Additional file 1: Supplementary Figure 1. Potential factors associate with DAM phenotypic transformation in epilepsy. (A) The differentially expressed genes (DEGs; red, upregulated genes; blue, downregulated genes) in the mouse model of epilepsy (Model, GSE1831 and GSE40490), TREM2 knockout (KO, GSE70475) group and DAP12 knockout (GSE9043) group relative to the control groups. DEGs were identified from pairwise comparisons with two selection criteria: fold change > 2.0 and corresponding adjusted P value < 0.05. (B) Venn diagrams of three commonly changed genes in four databases. (C) Heatmaps visualizing the mRNA expression of the three genes. [file 12974_2021_2133_MOESM1_ESM.tif]

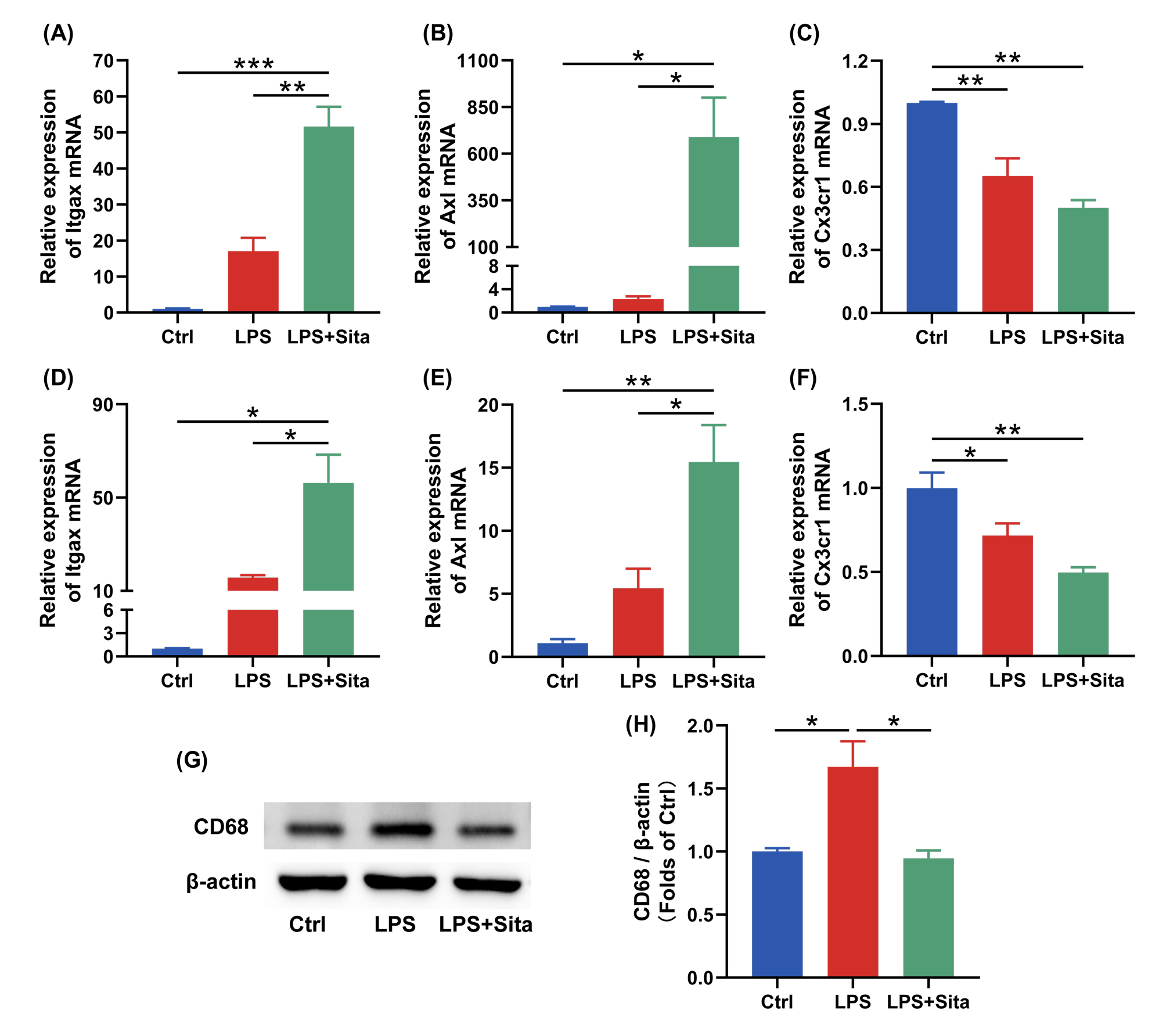

Supplement: Supplementary file 2 — Additional file 2: Supplementary Figure 2. Inhibition of DPP4 alters the activation state of microglia in vitro. (A–C) Itgax, Axl and Cx3cr1 mRNA expression was measured by RT-qPCR in both mouse primary microglial cells and (D–F) BV2 microglial cells. (G–H) Western blotting of CD68 protein in BV2 microglia. Relative CD68 expression was normalized to β-actin (n = 3). Data are presented as the mean ± SEM. *P < 0.05, **P < 0.01, ***P < 0.001. [file 12974_2021_2133_MOESM2_ESM.tif]

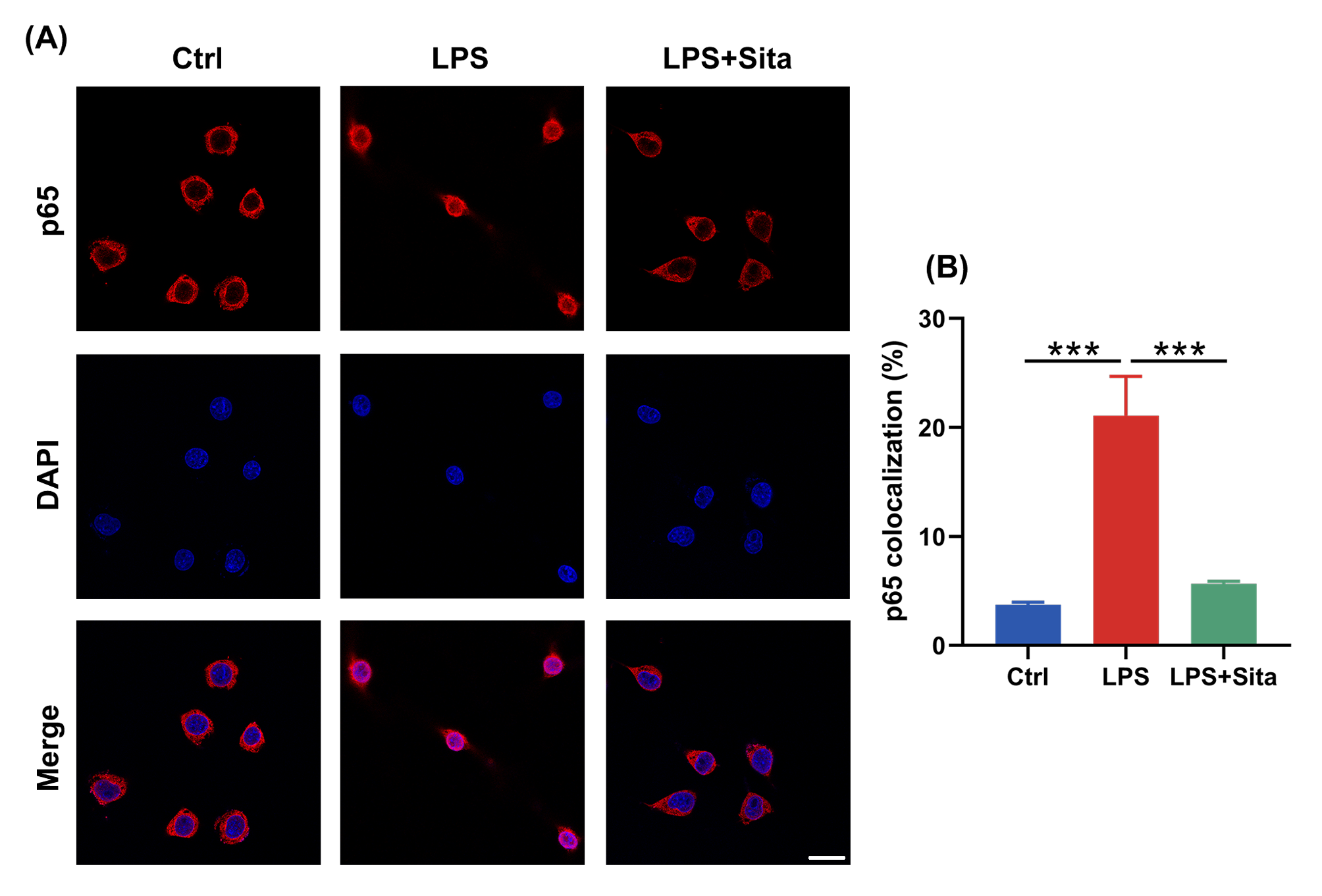

Supplement: Supplementary file 3 — Additional file 3: Supplementary Figure 3. Colocalization analysis of microglial cells. (A) Immunofluorescence staining showing that p65 colocalized with DAPI (magenta indicates a merge). (B) The statistical analysis is shown on the right side. Scale bar = 25 μm. Data are presented as the mean ± SEM. ***P < 0.001. [file 12974_2021_2133_MOESM3_ESM.tif]
