# Supplementary material for: The effect of dipeptidyl peptidase IV on disease-associated microglia phenotypic transformation in epilepsy
Source: J Neuroinflammation. 2021 May 11;18:112. doi: 10.1186/s12974-021-02133-y (PMC8114532; doi:10.1186/s12974-021-02133-y)
Supplement: Supplementary file 4 — Additional file 4: Supplementary Table 1. Primer sequence. [file 12974_2021_2133_MOESM4_ESM.docx]

Supplementary Table 1

Primer sequence.

| Gene | Upstream (5′–3′) | Downstream (5′–3′) |
| --- | --- | --- |
| Itgax | CCAAGACATCGTGTTCCTGATT | ACAGCTTTAACAAAGTCCAGCA |
| Cx3cr1 | GTTATTTGGGCGACATTGTGGC | CAGACCGAACGTGAAGACGAG |
| Axl | GGAACCCAGGGAATATCACAGG | AGTTCTAGGATCTGTCCATCTCG |
| GAPDH | AGGTCGGTGTGAACGGATTTG | GGGGTCGTTGATGGCAACA |
| IL-6 | CTGCAAGAGACTTCCATCCAG | AGTGGTATAGACAGGTCTGTTGG |
| IL-1β | GAAATGCCACCTTTTGACAGTG | TGGATGCTCTCATCAGGACAG |
| iNOS | GTTCTCAGCCCAACAATACAAGA | GTGGACGGGTCGATGTCAC |
| CD44 | TCTGCCATCTAGCACTAAGAGC | GTCTGGGTATTGAAAGGTGTAGC |
| Cxcr4 | GACTGGCATAGTCGGCAATG | AGAAGGGGAGTGTGATGACAAA |
