# Supplementary material for: The effect of dipeptidyl peptidase IV on disease-associated microglia phenotypic transformation in epilepsy
Source: J Neuroinflammation. 2021 May 11;18:112. doi: 10.1186/s12974-021-02133-y (PMC8114532; doi:10.1186/s12974-021-02133-y)
Supplement: Supplementary file 5 — Additional file 5: Supplementary Table 2. Datasets from GEO database. [file 12974_2021_2133_MOESM5_ESM.docx]

Supplementary Table 2

Datasets from GEO database.

| GEO accession number | Platform | Organism | Tissues | Pairwise comparisons |
| --- | --- | --- | --- | --- |
| GSE40490 | GPL4135 | Rattus norvegicus (5 weeks old) | brain, hippocampus | FeCl_3_ injection vs. Control (4 vs.4) |
| GSE1831 | GPL85 | Rattus norvegicus (2 weeks old) | brain, hippocampus | Kainate injection vs. Control (3 vs.3) |
| GSE70475 | GPL10740 | Mus musculus  (6 weeks old) | microglia | TREM2-Knockout vs. TREM2-Wild Type (4 vs.3) |
| GSE9043 | GPL1261 | Mus musculus (-) | microglia | DAP12^*^-Knockout vs. DAP12-Wild Type (3 vs.3) |

*DNAX-activation protein 12 (DAP12) is the ligand of TREM2 in microglia.
